# Utilization of lasso peptides for biodegradation of polycyclic aromatic hydrocarbons

**DOI:** 10.1111/1758-2229.13197

**Published:** 2024-04-10

**Authors:** Waltena Simpson, Robin L. Brigmon, Daniel Howard, Makaela Jackson, Alex Kugler, Victoria Brown

**Affiliations:** ^1^ Department of Biological and Physical Sciences South Carolina State University Orangeburg South Carolina USA; ^2^ Savannah River National Laboratory Aiken South Carolina USA

## Abstract

Many microbial genes involved in degrading recalcitrant environmental contaminants such as polycyclic aromatic hydrocarbons (PAHs) have been identified and characterized. However, all molecular mechanisms required for PAH utilization have not yet been elucidated. In this work, we demonstrate the proposed involvement of lasso peptides in the utilization of the PAH phenanthrene in *Sphingomonas* BPH. Transpositional mutagenesis of *Sphingomonas* BPH with the miniTn5 transposon yielded 3 phenanthrene utilization deficient mutants, #257, #1778, and #1782. In mutant #1782, Tn5 had inserted into the large subunit of the naph/bph dioxygenase gene. In mutant #1778, Tn5 had inserted into the B2 protease gene of a lasso peptide cluster. This finding is the first report on the role of lasso peptides in PAH utilization. Our studies also demonstrate that interruption of the lasso peptide cluster resulted in a significant increase in the amount of biosurfactant produced in the presence of glucose when compared to the wild‐type strain. Collectively, these results suggest that the mechanisms *Sphingomonas* BPH utilizes to degrade phenanthrene are far more complex than previously understood and that the #1778 mutant may be a good candidate for bioremediation when glucose is applied as an amendment due to its higher biosurfactant production.

## Graphical abstract

Here, we provide the first correlation of the inactivation of a lasso peptide processing gene with an inability to utilize a polycyclic aromatic hydrocarbon.

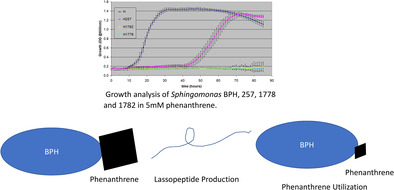

## INTRODUCTION

Polycyclic aromatic hydrocarbons (PAHs) are environmental contaminants that are toxic to human health as well as to the integrity of the soil, sediments, and/or groundwater. Phenanthrene is a three‐benzene ring fused angular PAH (Moody et al., 2001) which can be used as a sole carbon source by species such as *Pseudomonas, Burkholderia*, *Arthrobacter*, and *Sphingomonas* (Phale et al., 2019; Seo et al., 2009). Several pathways reported for phenanthrene degradation have been delineated (Doddamani & Ninnekar, 2000). In addition, the presence of a 232 kb genomic island (GEI) in *Delftia* sp. that encodes the genes for an entire phenanthrene catabolic pathway called the phn island has been reported (Hickey et al., 2012).

The biodegradation of PAHs is also impacted by biological surface‐active agents, known as biosurfactants. These amphipathic compounds reduce surface tension at the oil–water interfaces and can solubilize PAHs increasing their bioavailability (Y). Biosurfactants can facilitate the desorption of hydrocarbons from soil and sediment matrices, increasing bioavailability. Microbial biosurfactants are environmentally aesthetic due to their low‐cost, decreased invasiveness, production on demand, and minimal environmental toxicities as compared to inorganic surfactants (Franzetti et al., 2006). One of the main limitations for a wider application of inorganic surfactants in soil remediation is the lack of knowledge about the environmental fate and toxicity of the surfactant itself especially for in situ application. Sorption behaviour, biodegradability, toxicity of parent compound, and byproducts are essential processes that affect the environmental fate of surfactants in remediation applications. Desorption of PAHs from the soil or sediment matrices transfers the contaminants into the organic‐phase liquid, which is composed mostly of water‐insoluble organics (Christofi & Ivshina, 2002). Biosurfactants have been successfully applied in microbially‐enhanced oil recovery, cleaning oil‐contaminated vessels, and pipelines transporting heavy crude (Carrillo et al., 1996; Reis et al., 2013). Naturally occurring bacteria that produce biosurfactants provide an environmentally viable remedial option in concert with enzymatic activity.

A bacterial consortium of microorganisms designated BioTiger™ was isolated from waste lagoons of the Czechowice Oil Refinery (CZOR) in Poland (Reddy et al., 2020). Two members of the consortium include novel strains of *Sphingomonas*, designated BPH and BPF. In addition to producing biosurfactants, *Sphingomonas* strain BPH is capable of converting indole to indigo (indicating the presence of a dihydroxylating dehydrogenase gene), and degrading naphthalene, phenanthrene, fluorene, acenaphthene, fluoranthene, pyrene, and benzo[b]fluoranthene. To fully understand how the novel *Sphingomonas* strains accomplish their tasks, genes involved in PAH degradation must be identified. This will allow us to determine the extent to which BioTiger™ can be used in bioremediation.

Lasso peptides are a type of ribosomal natural product called RiPPs (ribosomally synthesized and posttranslationally modified peptides) which have been identified using genome mining and bioactivity screens in Proteobacteria (Chekan et al., 2016; Kodani et al., 2018; Maksimov et al., 2012; Zhu et al., 2016). Particular interest is focused on their antibacterial properties and ability to function therapeutically. Interest in lasso peptides has increased substantially due to their diverse functions (23) including receptor antagonists and enzyme inhibition. The archetype of lasso peptides is microcin J25, which was isolated from *E. coli* (Salomon & Farias, 1992). For microcin J25, the gene organization includes (1) *mcjA*, which encodes the lasso precursor peptide, (2) *mcjB*, which cleaves off the 37 amino acid leader peptide, thereby resulting in a 21 amino acid core, (3) *mcjC*, which is involved in the macro cyclization of the core peptide, and (4) *mcjD*, an ABC transporter involved in the export of the cyclized peptide (Kuroha et al., 2017; Zong et al., 2016).

Although many genes involved in PAH utilization have been identified and characterized, results in our laboratory indicate that the microbial mechanisms employed in this process are quite complex. While lasso peptides have been demonstrated to have varied functions, the literature does not contain prior reporting of their involvement in PAH utilization. In this report, we correlate the effect of a mutation of a lasso peptide with the inability of *Sphingomonas* to degrade phenanthrene. We hypothesize that our results suggest a role for lasso peptides in PAH utilization by a yet‐to‐be‐elucidated mechanism.

## EXPERIMENTAL PROCEDURES

### Bacterial strains and growth conditions

*Sphingomonas* BPH (ATCC accession number 5574) and *Escherichia coli* S17 lpir strains utilized in this study were maintained on R2A (Difco) plates supplemented with 25 μg/mL of colistin and Luria Bertani (LB; Difco) plates, respectively. *Escherichia coli* S17 l*pir*, harbouring the conjugal plasmid pUT:miniTn5KmluxABE, was maintained on LB plates supplemented with 100 μg/mL of kanamycin. *Sphingomonas* BPH PAH‐utilization mutants were routinely maintained on R2A plates supplemented with 100 μg/mL kanamycin and 25 mg/mL colistin.

### DNA isolation, sequencing, and analysis

DNA from *Sphingomonas* BPH mutants #1778 and #1782 was prepared as described in Simpson et al., 1999 and submitted to Georgia Genomics and Bioinformatic Core (Athens, GA). The samples were spiked into an Illumina MiSeq, PE150 run. The DNA libraries were NGS‐DNA, PCR Free. A KAPA (Roche) library kit was utilized and standard pre‐sequencing QC was done on the libraries, followed by Qubit analysis and qPCR. Both mutant genomes were assembled using Illumina short‐read data. As both 1778 and 1782 had been mutated via Tn5 transposition (which contains the kanamycin gene) the GenBank fasta file of pUTKm1(accession AF102233.1). Two blast databases were created, one for each of the assembly fastas that contained contigs ≥1 kb length and that had been previously ordered against the reference genome using Mauve. To determine which contigs contained the Kan gene sequence, a BlastN search was performed using the pUTkm1 sequence to query against the two blast databases the data were output in both tabular and standards blast alignment (.blastn) formats. A single contig was identified for each assembly that contained 100% identity across 1520 bases with no gaps. This sequence was identified as the kanamycin gene (aminoglycoside 3′‐phosphotransferase). The identified contigs and alignment regions for each strain were as follows: #1778: NODE_10, bases 65,197‐66,716; #1782:NODE_18, bases 7566‐9085. BankIt sequence accession number for *spg*B is WEA85824.

### Transpositional mutagenesis

For conjugation studies, *E. coli* S17 *lpir* (harbouring pUTminiTn5KmluxABE) was grown in 20 mL of LB broth supplemented with 100 μg/mL kanamycin shaking at 37°C until it reached an O.D._600_ of ~0.5 *Sphingomonas* strain BPH were grown in 40 mL of LB broth (shaking at 28°C) until an O.D._600_ of 0.5 was obtained. A liquid loopful of the *E. coli* culture was line streaked onto an R2A agar plate. The *Sphingomonas* cultures were centrifuged, the supernatant discarded, and the pellet resuspended in ~3 mL of LB. A liquid loopful of concentrated *Sphingomonas* culture was then line streaked adjacent to the *E. coli* line streak. The R2A agar plate was then incubated overnight at 28°C. The two‐line streaks were then mixed using a glass rod and incubated at room temperature overnight to allow conjugation to occur. The conjugation mix from the plates was then scraped and placed into 1 mL of 1× PBS (phosphate‐buffered saline, pH 7). Then 100 μL was removed and plated onto R2A agar plates supplemented with kanamycin (125 μg/mL) and colistin (25 μg/mL). The resulting transconjugants were then removed from the plates using sterile toothpicks and placed onto R2A plates supplemented with the antibiotic kanamycin and colistin (25 colonies per plate). The plates were then set out at room temperature and allowed to grow. Replica plating was subsequently performed on R2A plates supplemented with 100 μg/mL kanamycin.

### Southern blot analysis

To verify the presence of Tn5 in the PAH‐utilization mutants, Southern blot analysis was performed. Genomic DNA was isolated from the wildtype and mutant strains using the Ultra Clean Microbial DNA Isolation Kit (Mo Bio Laboratories, Solana Beach, CA). Genomic DNA (3 μg) was double digested with *Nde*I and *Aat*II (New England Biolabs, Beverly, MA) and electrophoresed on a 1% agarose gel. The gel was subsequently blotted against the N^+^ nylon membrane as described by Sambrook et al. Prehybridization of the membrane was performed at 68°C for 1 h using a prehybridization solution. The complete 816 bp kanamycin gene was PCR amplified from pUT: miniTn5KmluxAB (forward primer 5′ ATGAGCCATATTCAACG 3′; reverse primer 5′ TTAGAAAAACTCATCGAGC 3′; MoleculA, Inc, Columbia, MD). PCR amplification conditions: denaturation at 94°C for 1 min, annealing at 54°C for 1, extension at 72°C for 2 min. The PCR product was then excised from a 1% agarose gel and purified using the Wizard SV Gel and PCR Clean Up System (Promega Corp., Madison, WI). The probe was biotin labelled (dATP) as described by the NEBlot Phototope Kit (New England Biolabs, Beverly, MA). After prehybridization, the biotinylated kanamycin gene probe was denatured and added to the hybridization solution. Hybridization of the probe to the membrane was carried out overnight at 68°C. After hybridization, membranes were washed twice with 2× SSC‐0.1% SDS at room temperature, then washed twice with 0.1× SSC‐0.1% SDS at 68°C. Following the washes, chemiluminescent detection of the probe was performed using the Phototope‐Star Detection Kit (New England Biolabs, Beverly, MA). The membranes were then exposed to x‐ray film.

### Phenanthrene conversion screening

The transconjugants were screened for the inability to degrade phenanthrene. Transconjugants were picked from the original plate using a sterile toothpick, and replicas were plated onto R2A plates and allowed to grow overnight. They were then overs prayed with a 2.5% phenanthrene solution (made in acetone: hexanes 1:1) and incubated for 7–10 days at 28°C. A zone clear of crystals near the colonies was used to indicate phenanthrene conversion.

### Growth rate screening

*Sphingomonas* BPH wildtype and mutant cultures were grown to early stationary phase in 0.05% peptone, 0.1% glucose, 0.05% tryptone, 0.1% yeast extract (YE) with 6 g/L MgSO_4_ and 0.7 g/L CaCl_2_. Cultures were grown, shaking at room temperature with and without 50 μg/mL of Kanamycin, depending on the experimental design. Ten microliter (10 μL) aliquots of cultures with equivalent cell densities were used to inoculate 190 μL of the same medium containing when indicated 1% YE, and/or 1 and 5 mM phenanthrene delivered in acetone. Cells were washed with Phosphate Buffered Saline (PBS) at pH 7. Cultures with no PAH were amended with acetone to control for any biological effect it may have had on the experiments. Eight to 10 replicates of each culture condition were grown in a 100‐well honeycomb plate compatible with the Bioscreen C edition turbidity indicator (OY Growth Curves AB Ltd.). The honeycomb plates containing the cultures were incubated in the Bioscreen C incubator for approximately 84 h collecting data every hour at 26°C with medium intensity shaking.

### Examination of biosurfactant production

To determine the biosurfactant production of the bacteria of interest *Sphingomonas* BPH, 1778, 1782, and *Bacillus cereus* (ATCC 14579) were grown in both R2A media and R2A amended with 10 g/L glucose. Approximately 1 mL of 10^8^ cells were added to 100 mL of sterile media and cells were allowed to grow for 1 week at room temperature on a rotary shaker at 100 RPM. This method was adopted after Mukherjee et al., 2009. Samples were measured on a Shimadzu spectrophotometer UV‐2401PC (Kyoto, Japan). A standard curve was prepared using rhamnolipids (Sigma). Using the rhamnolipids working standard solution of 10 g/L was prepared and diluted to prepare standards ranging from 0.1 to 2 g/L. Standard was acidified with 6 N HCl (Fisher), to a pH of 2, and allowed to incubate in the dark at 4°C for 30 min. Samples were agitated using a vortexer and the optical density was taken at 600 nm using water as a reference. Standard points were prepared in duplicate. To measure the biosurfactant produced by the cell cultures samples were centrifuged at 10000*g* to pellet cells. The supernatant was collected and acidified and allowed to incubate as outlined previously. Precipitate biosurfactant was then pelleted via centrifugate at 10000*g*, resuspended in DI water, and measured at 600 nm using water as a reference. Cell densities were measured via OD_600_ in triplicate at the time of the biosurfactant sample collection.

## RESULTS AND DISCUSSION

### Production of phenanthrene utilization mutants

**FIGURE 1:**
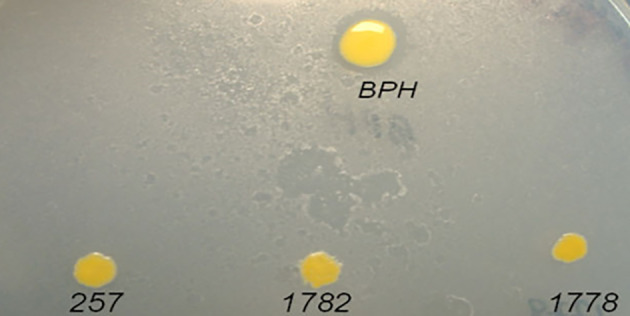
Phenanthrene (2.5%) overspray assay of *Sphingomonas* BPH (wildtype), and transconjugants 257, 1778 and 1782. Phenanthrene utilization is exhibited by the presence of a clear zone (halo) around the colony.

To identify new phenanthrene degrading genes, we initially performed transpositional mutagenesis of *Sphingomonas* BPH (recipient strain) and *E. coli* S17 *lair* harbouring the mini transposon miniTn5: kanluxABE. In the pUTminiTn5: kanluxABE plasmid, the genetic arrangement of transposase (*tnp*) is adjacent to, but outside of, the Tn5 transposon. This organization results in the loss of *tnp* following insertion and the stable inheritance of the mini‐transposon that is unlikely to cause DNA rearrangements or other forms of genetic instability (Lorenzo *et al*., 1998). Of the 2150 transconjugants produced, three (designated 257, 1778, and 1782) were demonstrated to be incapable of clearing phenanthrene crystals as determined by a 2.5% phenanthrene (acetone: hexanes 1:1) overspray assay (Figure 1). Incubation of the plates for a period of 8–10 days did not result in the clearing of phenanthrene crystals by the *Sphingomonas* mutants.

### *Utilization of phenanthrene as a sole carbon source by* Sphingomonas *mutants*

Growth analysis studies were performed to assess the abilities of the mutants to grow in liquid culture using 5 mM phenanthrene as a sole carbon source. Fresh cultures of BPH, 1778, 1782, and a set of control inactivated cells were washed 3× with sterile PBS were then spiked into Bushnell‐Haas medium with 5 mM phenanthrene to a concentration of 10^7–8^ cells/mL. Dead cells (Non‐viable controls) were obtained by autoclaving BPH.

**FIGURE. 2:**
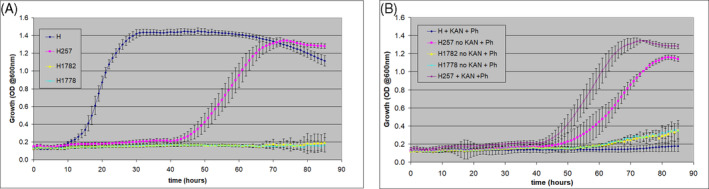
Growth analysis of *Sphingomonas* BPH, 257, 1778 and 1782 in 5 mM phenanthrene without kanamycin (A).

Briefly, phenanthrene was identified by an Agilent 6890 GC–MS in SIM mode (152, 176–179) equipped with a Restek 13,323 capillary column (30 m× 250 μm × 0.25 μm) as previously described (Reddy et al., 2020). Our results revealed transconjugant 257 exhibited a delayed ability to grow in the presence of phenanthrene (Figure 2A) and was not greatly affected by the addition of kanamycin (Figure 2B). In contrast, neither 1778 nor 1782 exhibited growth in 5 mM phenanthrene.

### *Identification of a putative* Sphingomonas *lasso peptide, sphingocin*

**FIGURE 3:**
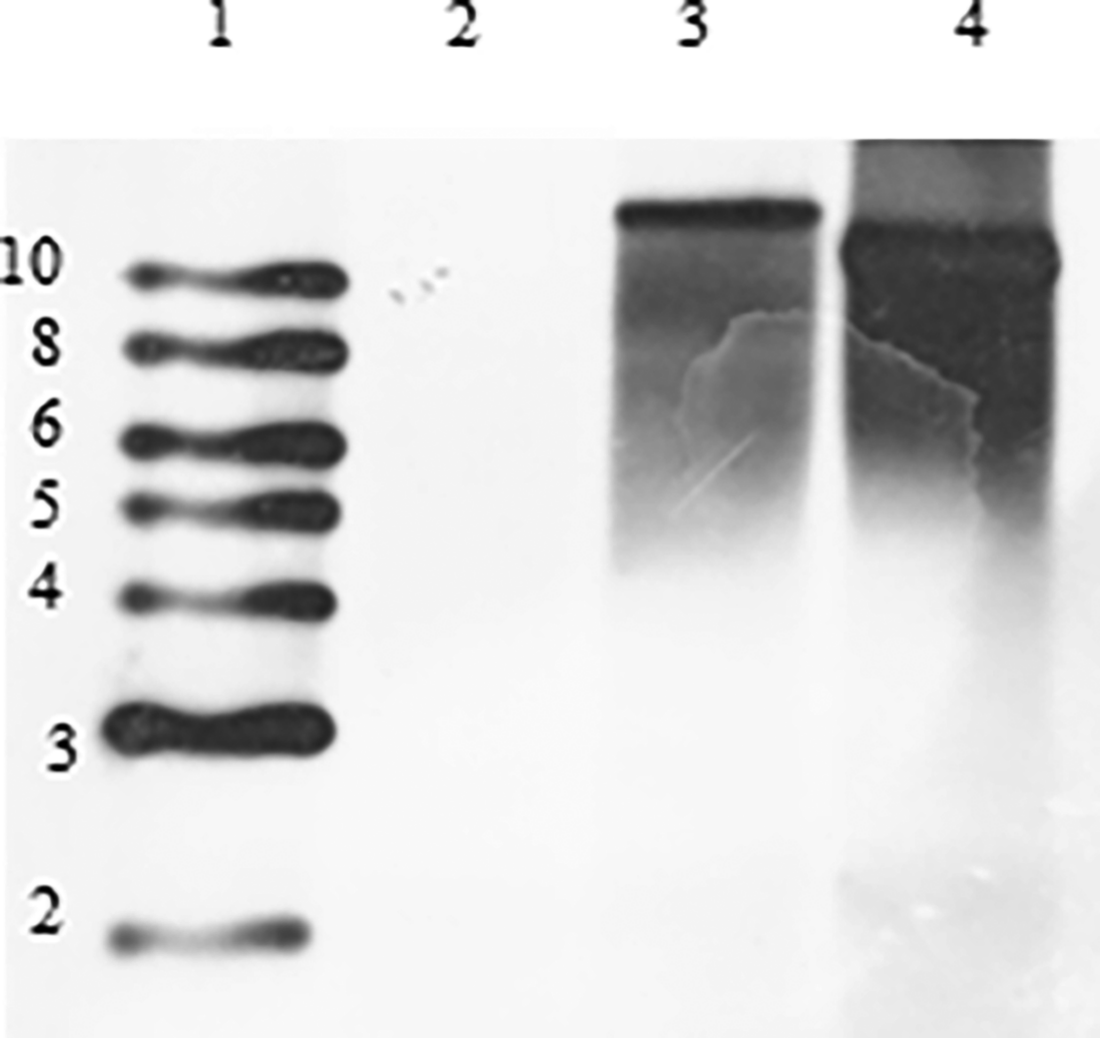
Southern Blot analysis of Sphingomonas 257, 1778 and 1782 using a kanamycin probe. Lane 1: 2‐ log Biotinylated ladder; Lane 2: *Sphingomonas* 257; Lane 3: *Sphingomonas* 1778; Lane 4: *Sphingomonas* 1782. Numbers to the left of the biotinylated ladder represent band sizes in kb.

Genomic analysis of *Sphingomonas* #257, #1778, and #1782 was then performed to determine the insertion site of the Tn5 transposon. Previous studies in our laboratory revealed the presence of a high molecular weight plasmid in the wildtype *Sphingomonas* strain (data not shown). As high molecular weight plasmids have been shown to harbour genes involved in PAH degradation, Southern blot analysis was performed to determine if the Tn5 insertion site was within the plasmid or genomic. To conduct this study, genomic DNA from strains #257, #1778, and #1782 was double digested with *Nde*I and *Aat*II (neither enzyme digests within the large Tn5:kmluxABE insert), transferred to a nylon membrane, and hybridized with PCR‐generated probe of the kanamycin resistance gene of the Tn5. Our results revealed no hybridization band in the #257 lane, indicating that the Tn5:luxABE transposon had inserted into this plasmid in this mutant. High molecular weight hybridization bands were observed in mutants #1778 and #1782 (Figure 3). Genomic DNA from strains #1782 and #1778 was isolated (Simpson et al., 1999) and subjected to nucleotide sequencing to identify the specific genes harbouring the miniTn5:kmluxABE insertion. Sequencing and analysis of genomic DNA were performed at the Georgia Genomics and Bioinformatic Core (Athens, GA).

**FIGURE 4:**
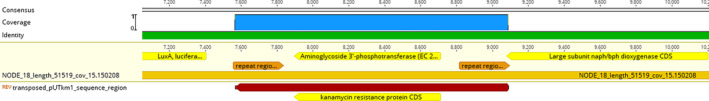
Insertion site of miniTn5Km:luxABE in *Sphingomonas* BPH 1782 into the large subunit of bphA1. The luciferase A gene, kanamycin resistance gene, and repeat regions of the transposon are indicated.

Examination of the insertion site in strain #1782 revealed that Tn5 had inserted into the large subunit of the naph/bph dioxygenase gene *bphA*1 (Figure 4), which initiates the catabolism of biphenyl via a deoxygenation reaction. This enzyme has been shown to have a broad substrate range, catalysing deoxygenation of 3 and 4‐ring PAHs, such as phenanthrene. The disruption of *bphA*1 directly correlates with the inability of mutant #1782 to degrade phenanthrene.

**FIGURE 5:**
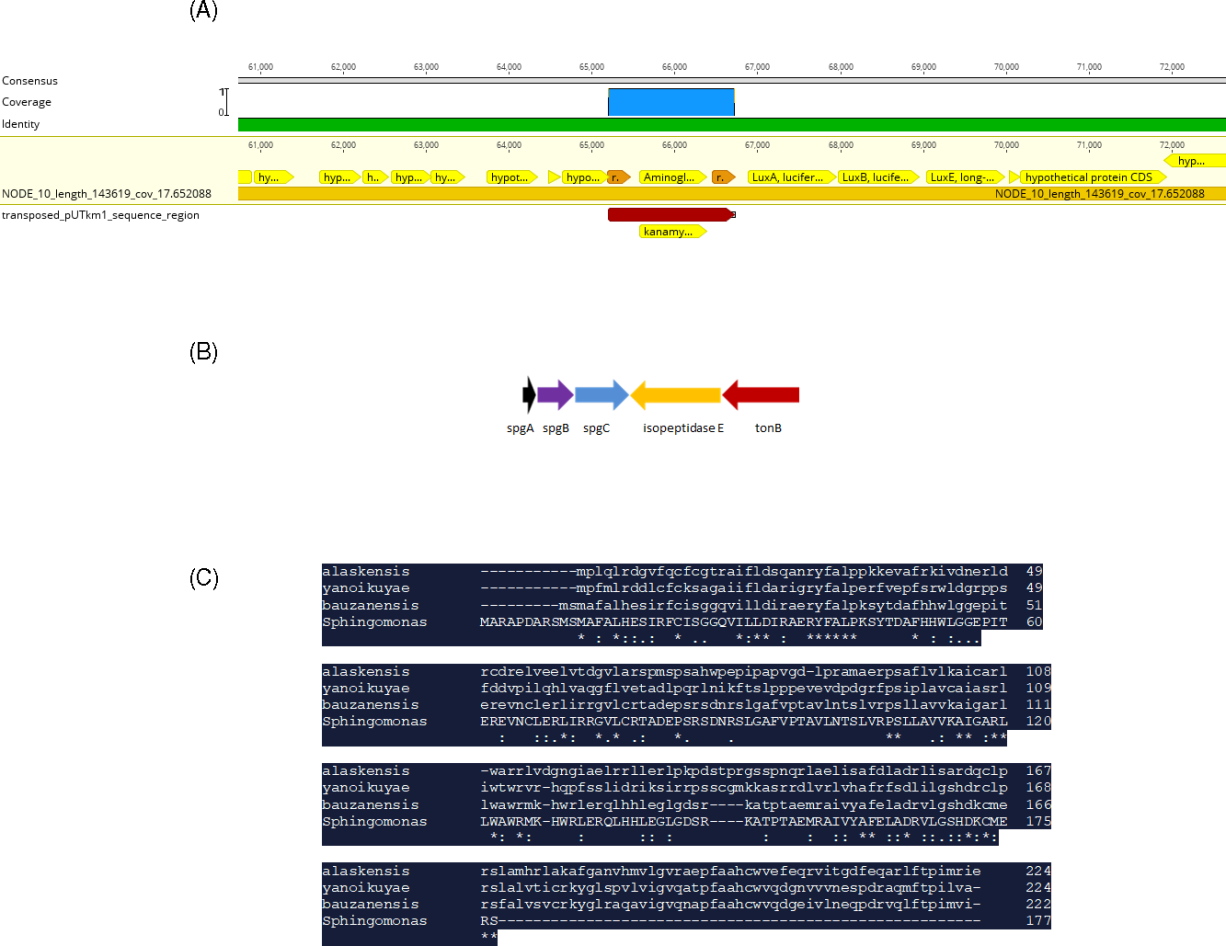
(A) Insertion site of miniTn5Km:luxABE transposon in *Sphingomonas* BPH 1778. Hypothetical protein coding regions surrounding the transposon represent the protease B2 gene of the lasso peptide operon. (B) Schematic representation of the *Sphingomonas* strain BPH sphingocin BCG. (C) Amino acid alignment between spgB of *Sphingomonas sp*. BPH and homologous B proteases from *Sphingopyzis alaskens*is, *Sphingobium yanoikuyae*, and *Sphingopyxis bauzanensis*. An asterik (*) indicates positions whish have a singly, fully conserved residue, a colon (:) indicates conservation between groups of strongly similar properties, and a period (.) indicates conservation between groups of weakly similar properties.

Examination of the Tn5 insertion site in strain #1778 revealed that Tn5 had inserted into a hypothetical protein, which when analysed, exhibited 100% identity to the lasso peptide B2 protein from *Sphingopyxis bauzanensis* (Figure 5A). The homology between *spg*B and *the lasso peptide B2* begins at amino acid 10 of *spg*B (Figure 5C). The B protease is required to bind and cleave the precursor lasso peptide, an integral part of the protein maturation process. The lasso peptide was designated sphingocin. Normally 15–24 amino acids long, the biosynthesis of the lasso peptide involves the formation of an isopeptide bond between the N‐terminal amine and the carboxy group of a glutamate or aspartate residue in the 7th, 8th, or 9th position, which gives rise to the macrolactam ring (Li et al., 2017; Maksimov et al., 2012). The C‐terminal linear peptide passes through the ring, thus giving rise to the “lasso” structure (Kodani et al., 2018; Maksimov et al., 2012). The lasso topology provides stability to the peptide, and many are resistant to proteolysis, chemical degradation, and high temperatures.

In our studies, analysis of the region surrounding the interrupted B2 protease identified a biosynthetic gene cluster (BGC) containing a lasso peptide operon and determined its organization. Our results indicate that the sphingocin operon consists of three genes: *spgA*, which encodes a 45 aa precursor protein (MNKELDHRDDELIDLGSVTEETKGPGFINNDGVGGKLPFAGLSDD*)*; *spgB*, which encodes a B2 protease; and *spgC*, which encodes an asparagine synthetase involved in macro cyclization of the core lasso peptide (Figure 5B). The accession number for *spg*B is WEA85824.

Interestingly, a D gene, which typically encodes for an ABC transporter, is not associated with this operon. The *spgA* gene exhibits 100% amino acid (aa) identity with a benenodin family lasso peptide from *S*. *bauzanensis*, while the *spgB* gene exhibits 100% aa identity with the protease B2 (lar2) of *S. bauzanensis*, 33% identity to the lasso B protein of *Sphingopyxis alaskensis*, and 37% identity to the lasso B protein of *Sphingobium yanoikuyae*. *spgC* exhibits 100% aa identity with the asparagine synthetase of *Sphingopyxis* sp. While many lasso peptides have an ABC transporter (D gene), lasso peptides devoid of a transporter have also been identified (Gomez‐Escribano et al., 2019; Hegemann et al., 2013; Maksimov et al., 2012). Although the amino acid sequence of the lasso peptide cluster of *Sphingomons sp* BPH is homologous to those previously identified, the insertion of Tn5 into the lasso B2 protein was of particular interest since it resulted in the loss of phenanthrene utilization. This is the first documentation of disruption of a gene involved in lasso peptide synthesis resulting in an inability to degrade a PAH such as phenanthrene.

Analysis of the SpgA precursor protein using RiPPMiner (http://www.nii.ac.in/rippminer.html) revealed a putative cleavage site to occur at amino acid 23, leaving a 22 aa core peptide (GPGFINNDGVGGKLPFAGLSDD). The first amino acid in the core peptide is glycine, as is typically seen with core lasso peptides. This model corresponds well with the typical 15–24 aa size of known lasso core peptides. Our results also indicate that a divergently transcribed set of genes is located adjacent to the sphingocin operon in *Sphingomonas* BPH. This set of genes consists of an isopeptidase (E) and a TonB‐dependent receptor. The gene encoding the isopeptidase was designated *spgE*. This specific organization of lasso peptides genes has been identified in the α‐Proteobacteria *Asticcacaulis excentricus* (Maksimov & Link, 2013), and *A. benevestitus* (Zong et al., 2017). In *A. excentricus*, the *atxE2* isopeptidase has been demonstrated to hydrolyze the lasso peptide and convert it into a linear form. As *spgE* exhibits 100% homology to *atxE2*, we hypothesize that it performs the same function in *Sphingomonas* BPH.

### *Examination of biosurfactant production in* Sphingomonas *BPH*

**FIGURE 6:**
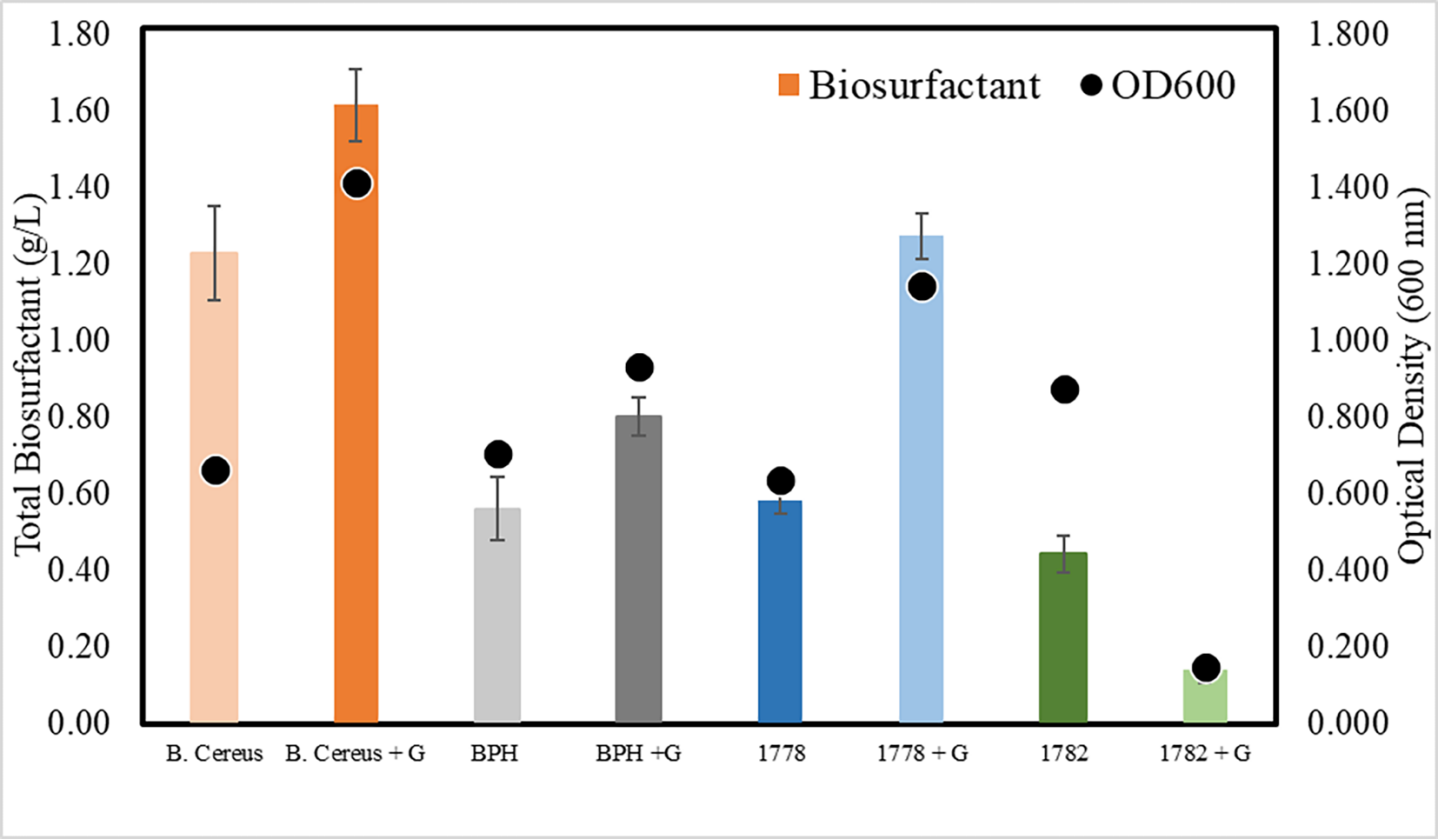
Total biosurfactant production in *B. cereus, Sphingomonas BPH, Sphingomona*s BPH #1778 and *Sphingomonas* BPH #1782. Cultures were grown in both R2A media and R2A amended with 10 g/L glucose. The method was adapted after Mukherjee et al., 2008. A standard curve was prepared using rhamnolipids (Sigma).

While the disruption of *spgB* resulted in the loss of the ability of the strain to degrade phenanthrene, the mechanism involved is unknown. It is well established that biosurfactants play a pivotal role in PAH degradation. Biosurfactants are capable of reducing interfacial and surface tensions, thereby increasing the bioavailability of environmental contaminants such as PAHs (Reddy et al., 2020). To determine if the *spgB* disruption affected biosurfactant production, we performed a quantitative total biosurfactant assay (Figure 6). *Bacillus cereus* is a known biosurfactant producer and used as a positive control, which produced over 1.2 g/L of total biosurfactant in the absence of glucose. The addition of glucose, as anticipated, increased the amount of total biosurfactant produced. In comparison, *Sphingomonas* BPH produced significantly less biosurfactant (approximately one‐half) than either positive control, regardless of growth condition. Interestingly, *Sphingomonas* BPH #1778 exhibited biosurfactant production comparable to that of the wild‐type strain in the absence of glucose. However, when amended with glucose, this strain produced significantly higher amounts of total biosurfactant as compared to the wild type. The mechanism responsible for this spike in total biosurfactant production, however, is currently unknown. *Sphingomonas* BPH #1782 displayed a slightly reduced level in the amount of total biosurfactant produced in the absence of glucose as compared to the wild type. In the presence of glucose, the total biosurfactant level was greatly reduced. This could be due, however to a low level of viability of the strain in the presence of glucose.

Here, we report on the first lasso peptide identified in *Sphingomonas* sp. BPH. While lasso peptides have been demonstrated to have antimicrobial, receptor antagonistic, and enzyme inhibitory functions, the literature does not contain prior reporting of their involvement in PAH utilization. While a lasso peptide cluster was recently found associated in a metagenomic assembled genome (MAG) of *Colwellia* which also contained subunits A and B of naphthalene dioxygenase enzyme, it was not hypothesized to be involved in naphthalene utilization in any way (Sieradzki et al., 2021). In this study, we have putatively identified a novel function of phenanthrene degradation for the lasso peptide cluster of *Sphingomonas* sp. BPH.

Most lasso peptide clusters have a D gene that encodes an ABC transporter believed to be responsible for secreting the peptide into the extracellular environment, however, several lasso peptide clusters do not. The absence of the D transporter does not always correlate with an inability of the peptide to be secreted into the supernatant. Interestingly, a recent study detailing the isolation of the lasso peptide subterisin of *Sphingomonas subterranea*, the BGC of which, while similar to *Sphingomonas* BPH, is devoid of an ABC transporter, demonstrated the presence of the peptide in the culture supernatant.

Studies in which lasso peptides are found in culture supernatants and those in which they are not have been reported. Analysis of *Streptomyces leeuwenhoekii* C34^T^ has revealed the presence of BGCs for three different lasso peptides (Gomez‐Escribano et al., 2019). Studies with the chaxapeptin lasso peptide from *S. leeuwenhoekii* C34^T^ revealed its detection in the culture supernatant, even though the chaxapeptin BGC does not include an ABC transporter. Contrastingly, the BGC for the novel leepeptin of *S. leeuwenhoekii* C34^T^ does contain an ABC transporter but is not detected in the supernatant (Gomez‐Escribano et al., 2019). The secretion of chaxapeptin after heterologous expression in *S. coelicolor* indicates that its export is mediated through a nonspecific secretion system in this strain. The lack of secretion of leepeptin into the culture supernatant, but secretion after heterologous expression in *S. coelicolor* may reflect a lack of activation of the regulatory mechanism required for leepeptin biosynthesis in *S. leeuwenhoekii* C34^T^. This indicates that certain environmental signals required for leepeptin production in *S. leeuwenhoikii* C34^T^ may not have been provided in laboratory experiments (Gomez‐Escribano et al., 2019).

Although sphingocin has not been isolated from *Sphingomonas* BPH, the results presented here allow us to postulate whether sphingocin would be present in the culture supernatant/lysate. The disruption of *sph*B and the resulting phenanthrene utilization phenotype indicates that the sphingocin cluster is not silent in *Sphingomonas* BPH but rather expressed. It also suggests that, similar to *S. leeuwenhoikii* C34^T^, a nonspecific secretion system may be involved in its secretion.

While the precise manner in which sphingocin disruption diminishes phenanthrene utilization has not yet been elucidated, our studies have established that it is not related to a decrease in biosurfactant production. Interestingly, a lack of sphingocin results in increased biosurfactant production in the presence of glucose. This suggests that *Sphingomonas* BPH #1778 may be a good candidate for bioremediation studies as it produces higher biosurfactant levels as compared to the wild type. Interestingly, it has been hypothesized that lasso peptide clusters that contain isopeptidases may function in a siderophore‐like manner. The isopeptidase is proposed to serve as a factor that releases cargo bound to lasso peptide (Chekan et al., 2016). The lasso peptide MccJ25 of *E. coli* has been shown to work in a “siderophore‐like” manner (Salomon & Farias, 1993; Salomon & Farias, 1995). Siderophores specifically bind/interact with the substances they transport and deliver them to the membrane. MccJ25 enters the target bacteria through the outer membrane siderophore receptor FhuA and inner membrane protein SbmA (Salomon & Farias, 1995). While it is unknown if the isopeptidase association with the sphingocin cluster functions as a siderophore for phenanthrene, this possibility warrants further investigation.

With the continued use of hydrocarbon fuels, ongoing disposal of toxic and recalcitrant PAHs into the environment remains a problem for now and in the future. The application of PAH‐degrading bacteria may provide a tool for environmental restoration and could serve as targets for biotechnologies. While monitoring and applying microorganisms capable of PAH remediation is important, optimization of the metabolic pathways requires knowledge of microbial ecology. More importantly, their molecular data remains an avenue for further development. This report suggests lasso peptides may play a significant role in PAH remediation in the environment. The combination of biodegradation data, and metabolic products, with metagenomics as presented here, could provide a source for the development of molecular tools and testing platforms for future clean‐up efforts.

## AUTHOR CONTRIBUTIONS

**Waltena Simpson:** Conceptualization (supporting); data curation (lead); formal analysis (lead); funding acquisition (lead); investigation (lead); methodology (lead); project administration (equal); resources (equal); software (supporting); supervision (lead); validation (supporting); visualization (supporting); writing – original draft (lead); writing – review and editing (supporting). **Robin L. Brigmon:** Conceptualization (supporting); data curation (supporting); formal analysis (equal); funding acquisition (supporting); investigation (equal); methodology (supporting); project administration (supporting); resources (equal); software (equal); supervision (supporting); validation (supporting); visualization (supporting); writing – original draft (supporting); writing – review and editing (equal). **Daniel Howard:** Conceptualization (supporting); data curation (supporting); formal analysis (supporting); funding acquisition (supporting); investigation (supporting); methodology (supporting); resources (supporting); software (supporting); supervision (supporting); validation (supporting); visualization (supporting); writing – original draft (supporting); writing – review and editing (supporting). **Victoria Brown:** Conceptualization (supporting); data curation (supporting); formal analysis (supporting); funding acquisition (supporting); investigation (supporting); methodology (supporting); project administration (supporting); resources (supporting); software (supporting); supervision (supporting); validation (supporting); visualization (supporting); writing – original draft (supporting); writing – review and editing (supporting). **Makaela Jackson:** Conceptualization (supporting); data curation (supporting); formal analysis (supporting); funding acquisition (supporting); investigation (supporting); methodology (supporting); project administration (supporting); resources (supporting); software (supporting); supervision (supporting); validation (supporting); visualization (supporting); writing – original draft (supporting); writing – review and editing (supporting). **Alex Kugler:** Conceptualization (supporting); data curation (supporting); formal analysis (supporting); funding acquisition (supporting); investigation (supporting); methodology (equal); project administration (supporting); resources (equal); software (supporting); supervision (supporting); validation (supporting); visualization (supporting); writing – original draft (equal); writing – review and editing (supporting).

## CONFLICT OF INTEREST STATEMENT

The authors declare no conflicts of interest.

## Data Availability

The author confirms that the data supporting the findings of this study are available in the article or its supplemental materials.
